# Reconstruction of Mouse Testicular Cellular Microenvironments in Long-Term Seminiferous Tubule Culture

**DOI:** 10.1371/journal.pone.0090088

**Published:** 2014-03-11

**Authors:** Juho-Antti Mäkelä, Jorma Toppari, Adolfo Rivero-Müller, Sami Ventelä

**Affiliations:** 1 Department of Physiology, University of Turku, Turku, Finland; 2 Department of Paediatrics, University of Turku, Turku, Finland; 3 Department of Otorhinolaryngology, Turku University Hospital, Turku, Finland; 4 Australian Regenerative Medicine Institute, Monash University, Clayton, Australia; University Hospital of Münster, Germany

## Abstract

Research on spermatogonia is hampered by complex architecture of the seminiferous tubule, poor viability of testicular tissue *ex vivo* and lack of physiologically relevant long-term culture systems. Therefore there is a need for an *in vitro* model that would enable long term survival and propagation of spermatogonia. We aimed at the most simplified approach to enable all different cell types within the seminiferous tubules to contribute to the creation of a niche for spermatogonia. In the present study we describe the establishment of a co-culture of mouse testicular cells that is based on proliferative and migratory activity of seminiferous tubule cells and does not involve separation, purification or differential plating of individual cell populations. The co-culture is composed of the constituents of testicular stem cell niche: Sertoli cells [identified by expression of Wilm's tumour antigen 1 (WT1) and secretion of glial cell line-derived neurotrophic factor, GDNF], peritubular myoid cells (expressing alpha smooth muscle actin, αSMA) and spermatogonia [expressing MAGE-B4, PLZF (promyelocytic leukaemia zinc finger), LIN28, *Gpr125* (G protein-coupled receptor 125), *CD9*, *c-Kit* and Nanog], and can be maintained for at least five weeks. GDNF was found in the medium at a sufficient concentration to support proliferating spermatogonial stem cells (SSCs) that were able to start spermatogenic differentiation after transplantation to an experimentally sterile recipient testis. *Gdnf* mRNA levels were elevated by follicle-stimulating hormone (FSH) which shows that the Sertoli cells in the co-culture respond to physiological stimuli. After approximately 2–4 weeks of culture a spontaneous formation of cord-like structures was monitored. These structures can be more than 10 mm in length and branch. They are formed by peritubular myoid cells, Sertoli cells, fibroblasts and spermatogonia as assessed by gene expression profiling. In conclusion, we have managed to establish *in vitro* conditions that allow spontaneous reconstruction of testicular cellular microenvironments.

## Introduction

Spermatogenic potential ultimately depends on a small population of spermatogonia called spermatogonial stem cells (SSCs). These cells are responsible for the life-long ability of sperm production in mammals and they are able to reconstitute spermatogenesis to recipients that are rendered experimentally sterile [1], [2]. Additionally, derivation of embryonic stem cell-like cells from SSCs has been demonstrated in a number of studies [3]–[5] and they hold thus a great promise for regenerative medicine by providing an ethically sustainable and immunologically competent source of pluripotent cells. A very small fraction of cells in SSC cultures behave like SSCs *in vivo* and are able to reconstitute spermatogenesis after transplantation [6]. Cells in SSC cultures are a heterogenous population and it is not clear how closely they resemble undifferentiated spermatogonia *in vivo*. Therefore their suitability to study germ cell biology can be justifiably questioned.

In the testis spermatogonia dwell on the basement membrane of the seminiferous epithelium. They are in physical contact with Sertoli cells and in close proximity of peritubular myoid cells. This is the physiologically relevant environment to study germ cell biology. Cell fate decisions of spermatogonia are thought to be directed by extracellular signals secreted by cells in their vicinity or carried to the testis by circulation. SSCs are maintained in the testis in specified areas, i.e. stem cell niches. There is a handful of molecular and anatomical cues about the location of testicular stem cell niche [7]–[11]. However, due to the complex architecture of the seminiferous tubules, *in vivo* studies on testis stem cell niche are limited and challenging. Moreover, there are not long-term organ cultures for adult testicular tissue to date. Fragments of rodent seminiferous tubules can be successfully cultured in defined conditions for up to 3–7 days [12]–[14], but already after a 16-hour culture a vast number of apoptotic cells can be observed [15] and the number increases rapidly within the next 2 days (Mäkelä J-A & Toppari J, unpublished data).

Recently, Sato and colleagues managed to produce functional male gametes *in vitro* using a neonatal mouse organ culture approach [16]–[17]. The same group demonstrated the progression of male germ cells at least up to meiotic prophase in *in vitro* reconstructed tubules of juvenile mouse testis cells [18]. Despite such progress in the field, their suitability to study dynamics of the adult stem cell niche is questionable. This is because adult SSCs are maintained in a quite distinct environment from that of the neonatal testis. In the adult testis somatic cells have seized proliferating and their secretory profile and mechanisms of regulation are different from the juvenile counterparts. Therefore new *in vitro* systems need to be developed to understand the regulation and function of adult testis stem cell niche.

The aim of the present study was to evaluate how well spermatogonia can be maintained in a seminiferous tubule cell-derived co-culture model. We hypothesized that co-culture of these cells would allow SSCs to survive and proliferate - something that SSCs cannot perform by themselves. Since the co-culture was maintained at 37°C and testosterone-producing Leydig cells did not contribute to it, we expected germ cells not to differentiate, the scope of the study being in early germ cell behaviour. We were able to support the survival and propagation of spermatogonia for at least 5 weeks and recorded relatively high levels of endogenous GDNF in the culture medium during that time. These data suggest that stem cell niche-like conditions had been reconstructed *in vitro*. Spermatogonia in the co-culture expressed a wide range of spermatogonial markers, including SSC markers PLZF (promyelocytic leukaemia zinc finger) [19], [20] and LIN28 [21], and undifferentiated spermatogonial marker Nanog [11] and they were able to colonize seminiferous tubules of busulfan-treated mice. Cells in the culture were also observed to give rise to secondary structures, i.e. spherical cell aggregates and cord-like structures. Therefore the present model might also be used to study early steps of development and regeneration of testis tissue *in vitro*.

## Results

### Establishment of the co-culture

**Figure 1:**
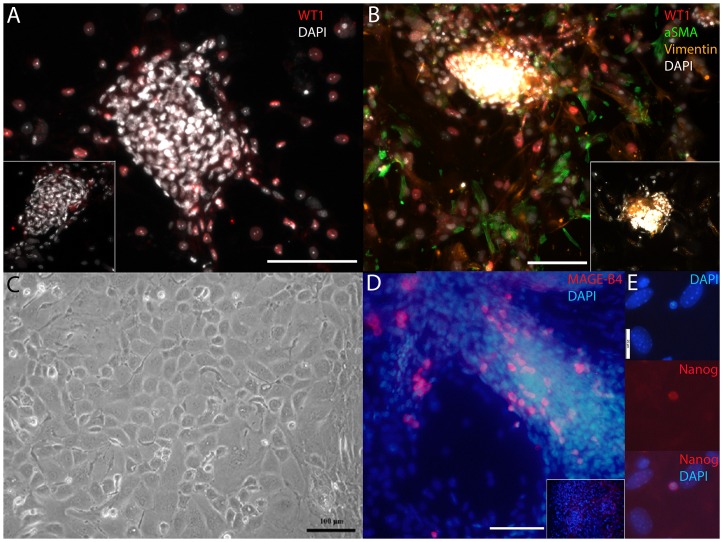
Early stages in the establishment of seminiferous tubule-derived co-culture. **A**) Five-day co-culture showing WT1 positive cells (red) in the perimeter of a seminiferous tubule fragment. **B**) WT1 (red), Vimentin (orange), Alpha smooth muscle actin (αSMA, green) immunocytochemical staining for 1-week co-culture. DAPI (white) stains the nuclei in A and B. DAPI dense areas are fragments of seminiferous tubule. **C**) Phase-contrast microscopy image of 1-week co-culture. **D**) MAGE-B4-expressing (red) cells in 1-week co-culture. **E**) An example of a rare Nanog-expressing cell in 2-week co-culture. DAPI stains the nuclei blue in D and E. Scale bar is 100 and 25 µm in A–D and E, respectively. Insets in **A**, **B** and **D** represent negative control stainings.

Mouse testes were decapsulated and the seminiferous tubules were separated from each other and from the interstitial tissue in sterile conditions in DMEM/F12 supplemented with 15% (v/v) inactivated fetal calf serum (iFCS) (referred to as culture medium later in the text). The tubules were cut into small fragments and 50–100 approximately 1-mm-long segments of mouse seminiferous tubule were pipetted onto a culture dish in small volume of culture medium (100, 200 and 350 µl of suspension was plated onto 24-, 12-, and 6-wells plates, respectively). After 6–10 hours of incubation more culture medium was added (500, 1000 and 1500 µl was pipetted onto 24-, 12-, and 6-wells plates, respectively). Five to seven days later we could observe spreading of WT1 (Wilm's tumour antigen 1)- [22] and Vimentin-expressing Sertoli cells and alpha smooth muscle actin (αSMA)-expressing peritubular myoid cells [23] in the perimeter of seminiferous tubule fragments (Figure 1A–B; Figure S1 for positive and negative control stainings for testicular tissue sections). After 1–2 weeks of culture, the seminiferous tubule cells had spread greatly and formed a nearly confluent monolayer culture (Figure 1C). Immunocytochemical analysis revealed that many cells in the co-culture expressed MAGE-B4 (Figure 1D), a ubiquitous spermatogonial cell marker [24] (Figure S1), while only a few cells expressed A-single undifferentiated spermatogonial cell marker Nanog [11] (Figure 1E, S2). Utilising the same technique, we successfully established this kind of co-cultures from juvenile, pubertal, adult and elderly mice. Adult-derived co-cultures are used in analyses throughout this report if not otherwise stated.

**Figure 2:**
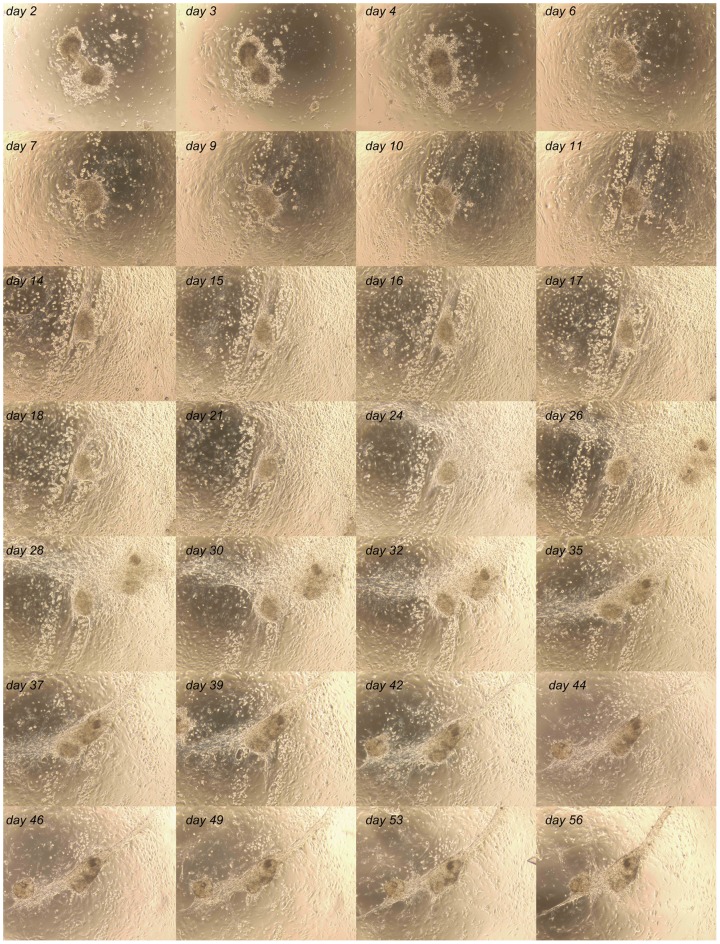
Eight-week follow-up of adult-derived seminiferous tubule cell co-culture. Cells proliferated rapidly to form a confluent culture. First signs of cord-like structure formation were apparent after four weeks of culture in this case.

We followed the co-culture up to 8 weeks and performed a time-lapse imaging series by photographing specific areas in the co-culture every 1–3 days. During this period of time we observed partly dissociation of the seminiferous tubule fragments (during the first week), gain of confluence within the first 1–2 weeks and formation of cord-like structures and cell clusters during weeks 2–6 (Figure 2 and Video S1). Formation of clusters and cord-like structures was associated with dramatic and dynamic changes in the interaction between different cell populations in the co-culture, whereas during the first 1–2 weeks the cells were mainly found single-dwelling or in small populations within the co-culture. In the co-culture at least two different testicular cellular microenvironments were reconstructed: 1. testis stem cell niche-like conditions, and 2. *in vitro* milieu enabling cluster and cord-like structure formation by testicular somatic cells.

### Maintenance and propagation of germ cells in the co-culture

**Figure 3:**
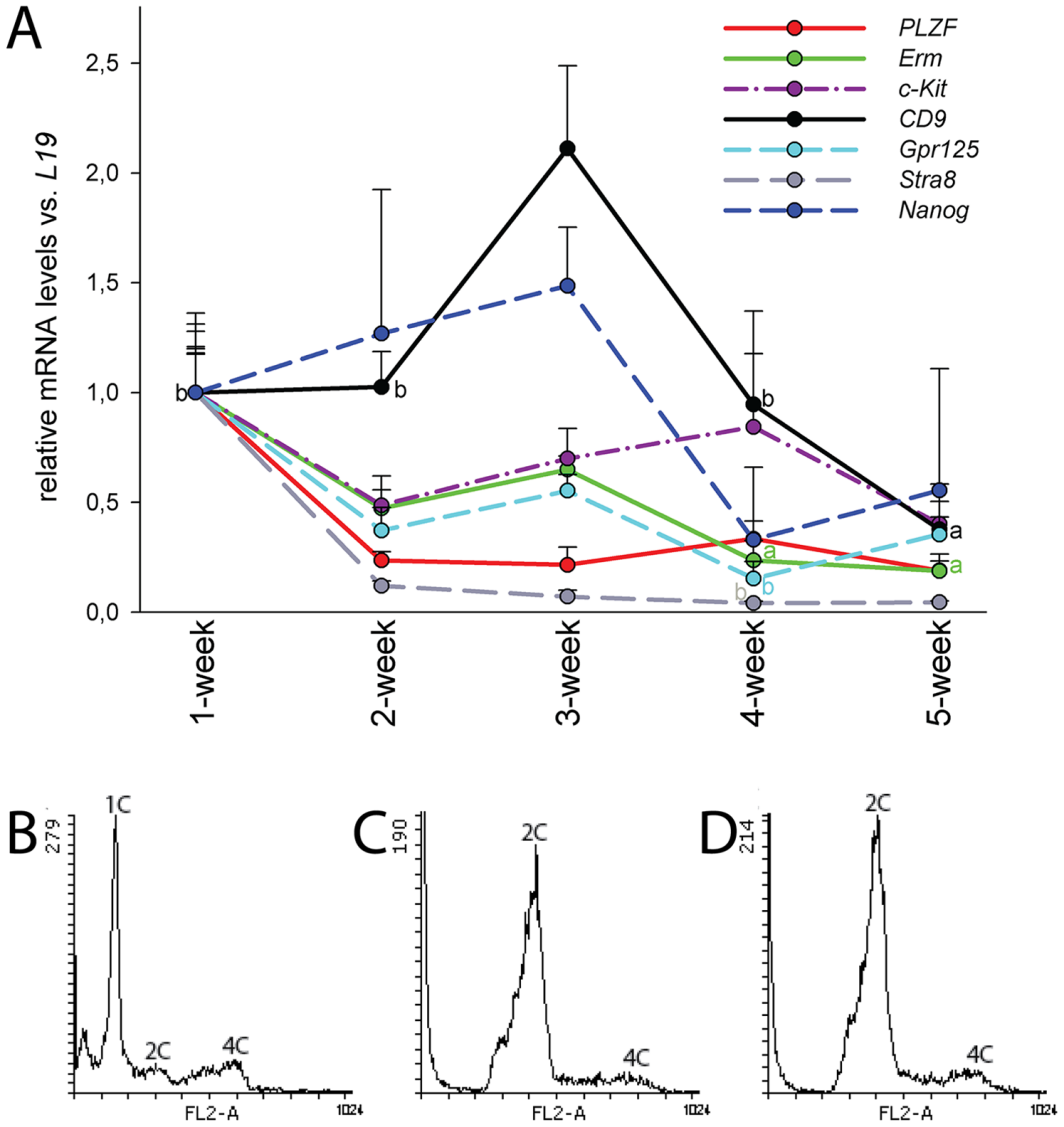
Messenger RNAs of spermatogonial markers are expressed in the co-cultures until 5 weeks, whereas haploid cells are lost during the first week of culture. **A**) Quantitative RT-PCR analysis of spermatogonial cell-associated markers during 1–5 weeks of culture. Steady state levels of *CD9* mRNA were at their highest at three weeks. *Erm*, *Gpr125* and *Stra8* levels decreased towards the end of the culture. There were no statistically significant changes for *c-Kit*, *Nanog* and *PLZF*. Statistical significances are reported in comparison with the time point of the highest value. n = 3–6, SEM; **a**, p<0.01; **b**, p<0.05. Colour-coding in letters “a” and “b” refers to the colour of the lines. **B–D**) Analysis of haploid and diploid cells in co-cultures. Nuclei from co-cultures were stained with propidium iodide and analysed by flow cytometry: **B**) Starting material, **C**) 1-week co-culture and **D**) 2-week co-culture. Haploid cells (1C) were lost during the first week of culture. Relative fraction of G0/G1 cells (2C) increased rapidly, whereas the relative number of G1S/G2/M cells (4C) first decreased but later on stayed at a stable level. Histograms are representative of at least three parallel experiments.

To find out which germ cell populations are maintained in the prevailing conditions we analysed the steady state levels of *CD9* (CD9 antigen) [25], *c-Kit* [26], *Erm* (Ets-related molecule) [27], [28], *Gpr125* [29], *Nanog*, *PLZF*, *Stra8* (Stimulated by retinoic acid gene 8) [30], [31] and *Sycp3* (Synaptonemal complex protein 3) [32] mRNAs throughout 5 weeks of culture. Because the full complement of germ cells is present only in the adult testis, we used seminiferous tubule segments from adult mice to establish the co-cultures for this experiment. We observed that *Gpr125*, *Erm* and *Stra8* mRNA levels slowly decreased with time until statistically significant differences were recorded at the late time points (Figure 3A). Transcript levels of *c-Kit*, *Nanog* and *PLZF* did not change in a statistically significant manner, whereas at 3 weeks the highest *CD9* mRNA levels were recorded. Transcript for meiotic cell marker *Sycp3* was not detectable after the first week (data not shown). These data suggest that spermatogonia survive in these culture conditions for many weeks, whereas more differentiated germ cells do not. This was also supported by propidium iodide (PI) staining of cell nuclei followed by flow cytometry showing that while haploid cells represented by far the greatest fraction in the beginning of the culture (Figure 3B) they virtually vanished after the first week. Most of the nuclei from then on were from cells in G0/G1 phase (Figure 3C–D). Loss of haploid cells is due to the 37°C temperature and perhaps also due to lack of testosterone: we were able to detect measurable but hypophysiological [33] levels of testosterone in 1–2 and 1-week cultures started from adult and juvenile mice, respectively (Table 1). Thereafter the levels fell below the sensitivity range of the used assay. Stimulation of 2–3-week cultures with an LH/hCG analogue did not increase the levels of testosterone in the medium (data not shown) proving the absence of Leydig cells in the co-cultures after 1–2 weeks.

**Table 1:** Testosterone concentration in the culture medium.

	1-week (n = 3)	2-week (n = 3)	3–4-week (n = 6)
Co-culture started from seminiferous tubules of adult mice	1,79±0,24 nmol/l	0,87±0,03 nmol/l	Measured values below the sensitivity range of the assay

	1-week (n = 4)	2–5-week (n = 16)
Co-culture started from seminiferous tubules of juvenile mice	0,87±0,73 nmol/l	Measured values below the sensitivity range of the assay

The values represent average ± standard deviation.

**Figure 4:**
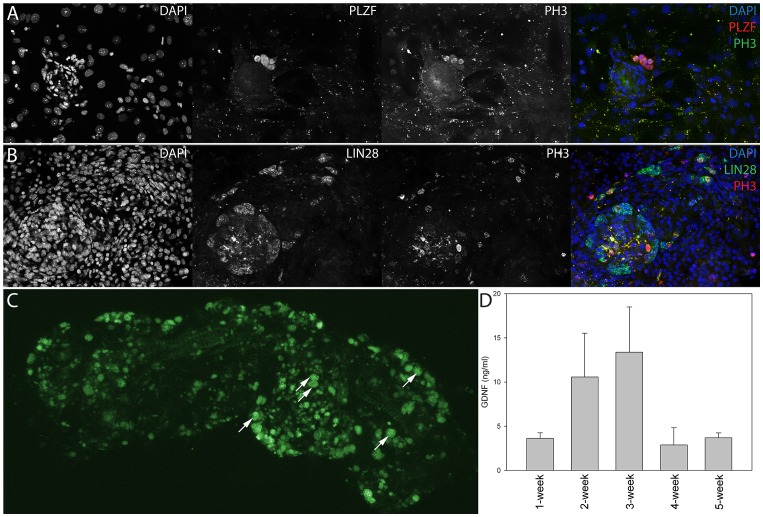
Spermatogonial stem cells were identified in co-cultures by immunocytochemistry and transplantation assay, and these cells were supported by endogenous GDNF secretion. **A**) Image of 1-week-old co-culture showing proliferating, phosphorylated Histone-3 (PH3) positive cells expressing spermatogonial stem cell marker PLZF. **B**) A high proportion of LIN28 positive cells also expressed PH3 after 10 days of culture. DAPI stains the nuclei of cells. **C**) Section of a wild-type SSC-depleted seminiferous tubule that has been colonized by transplanted SSCs. Samples were prepared five weeks after transplantation of GFP-expressing SSCs and show that spermatogenic differentiation of the transplanted SSCs had advanced to meiotic prophase. Arrows indicate pachytene spermatocytes. **D**) GDNF concentration in co-cultures. Analysis of GDNF levels in the culture medium showed that approximately 5–15 ng/ml were present in 1–5-week co-cultures.

Spermatogonia expressing SSC-associated proteins PLZF, LIN28 and ubiquitous germ cell marker Ddx4 [34] were found to be proliferatively active as judged by the coexpression of proliferating cell antigens PH3 (phosphorylated Histone-3) and Ki-67 (Figure 4A–B, Figure S3). Generally, cells expressing spermatogonia-associated markers were usually located at areas of high cell density (Figure 1 and 4). These data suggest that at those areas the microenvironment was favourable for spermatogonial cell survival and propagation and provided testis stem cell niche-like conditions for SSCs.

After noting that the co-culture conditions were favourable for spermatogonia expressing SSC-associated markers, we wanted to study whether SSCs in the co-culture maintained their differentiation potential. For this purpose we established the co-cultures from adult GFP-expressing mice seminiferous tubules as described above. Then we treated adult wild-type male C57BL/6 mice with busulfan (40 mg/kg, i.p.) which results in the depletion of SSCs [35]. One month after the busulfan injection approximately 100 000 cells from 1-month-old co-cultures were transplanted via the rete testis according to a protocol described previously [36]. Five weeks later mice were sacrificed and seminiferous tubules were dissected and imaged under a fluorescence Zeiss SteREO Lumar V12 stereomicroscope with eGFP bandpass filter. The presence of GFP-positive pachytene spermatocytes inside seminiferous tubule segments indicated that the transplanted cells had started to undergo spermatogenic differentiation *in vivo* (Figure 4C). These data show that SSCs can be maintained in an undifferentiated state in the co-culture for at least a month. This was probably due to secretion of Sertoli cell-derived GDNF which has been shown to be crucial for SSC viability both *in vivo* and *in vitro* [37]. Using an ELISA-GDNF assay we detected 5–15 ng/ml of GDNF in the culture medium during 1–5 weeks of culture (Figure 4D), concentrations that are in the range of those that are commonly used in SSC cultures where GDNF is added to the culture medium as a supplement [6]. Addition of exogenous GDNF (10 ng/ml) to culture medium did not result in morphological alterations or dramatic changes in the number of spermatogonia in culture. At transcript level only the relative amount of *CIP2A* (cancerous inhibitor of PP2A) [38]–[39] mRNA was elevated (data not shown) by including exogenous GDNF in the culture medium.

**Figure 5:**
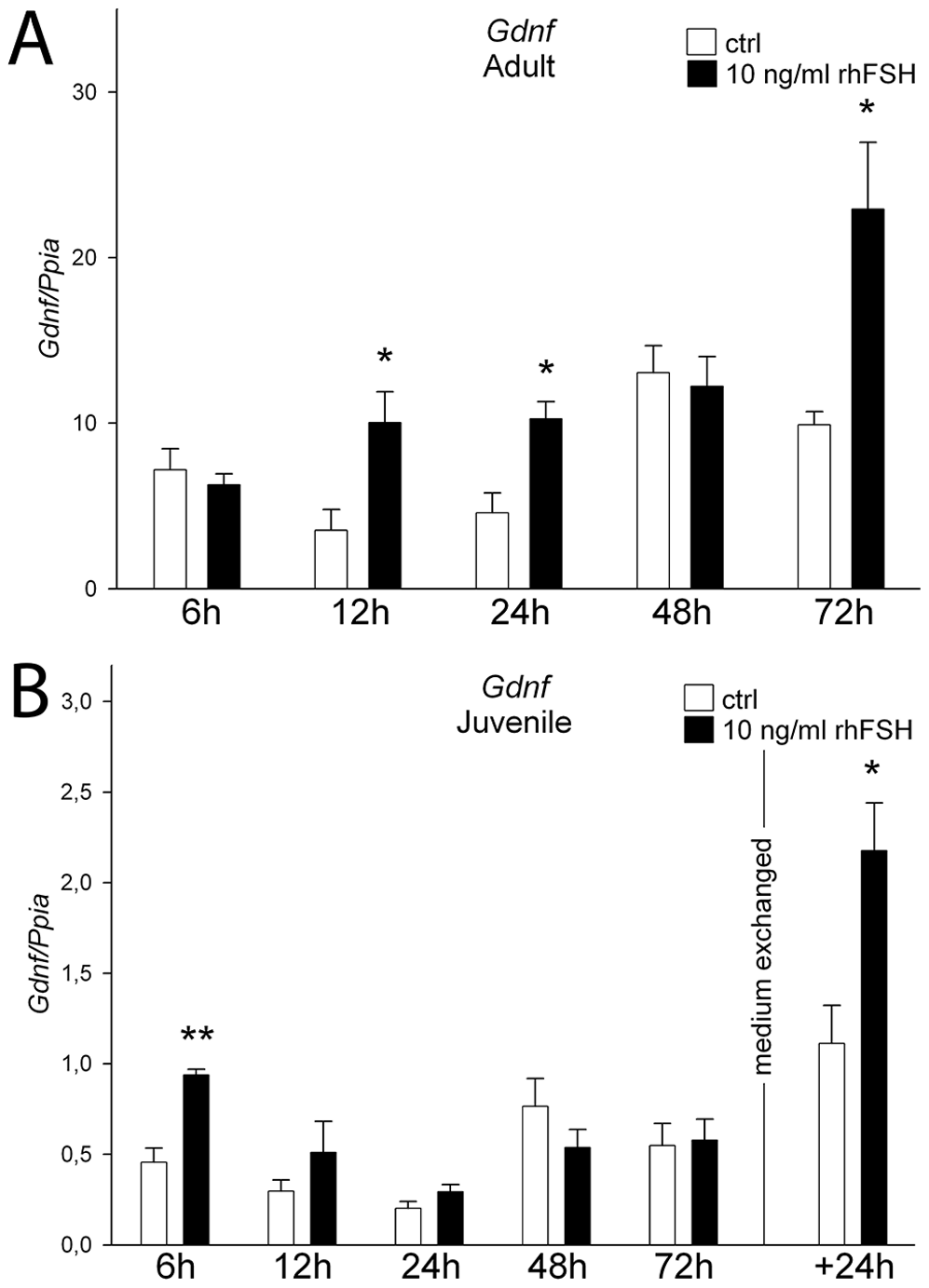
Co-cultures responded to FSH. Recombinant human FSH treatment resulted in elevated *Gdnf* mRNA levels in 1-week co-cultures started from **A**) adult and **B**) juvenile seminiferous tubule fragments. In juvenile mouse-derived co-cultures FSH treatment was followed by an acute increase in *Gdnf* mRNA levels at 6 hours. Retreatment 72 hours after initial dose lead to increased levels 24 hours later. White bars, control; black bars, 10 ng/ml rhFSH; n = 3, SEM; *, p<0.05; **, p<0.01.

To study if Sertoli cells in the co-culture respond to physiological stimuli, we treated 1-week-old co-cultures with recombinant human follicle-stimulating hormone (rhFSH; 10 ng/ml) for 6, 12, 24, 48 and 72 hours. The treatment increased the steady state levels of *Gdnf* mRNA 12, 24 and 72 hours after the treatment (Figure 5A). Moreover, FSH also stimulated the levels of *Gdnf* mRNA in the co-cultures that were established from juvenile (<10-day-old) mice (Figure 5B) albeit in a more acute fashion. FSH treatment also secondarily affected the steady state levels of some spermatogonia-associated mRNAs, most clearly those of *Gfr1a* and *Stra8* (Figure S4).

### Formation of clusters and cord-like structures in vitro

**Figure 6:**
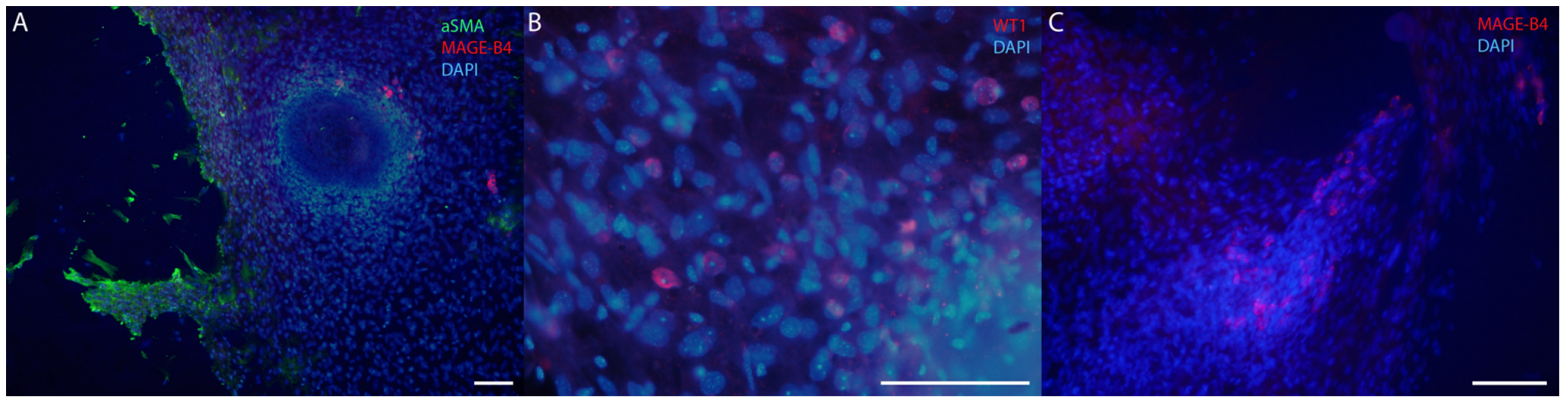
Peritubular myoid, Sertoli and spermatogonial cells are situated differently when secondary structure formation starts. **A**) Alpha smooth muscle actin (green) positive cells were found at the frontier of a tightening cell network. **B**) Sertoli cells (WT1, red) were situated at areas of lower cell density, whereas **C**) spermatogonia (MAGE-B4, red) were found in clusters or single-dwelling at high cell density areas. There are MAGE-B4 positive cells (red) in **A**), as well. DAPI (blue) stains the nuclei. Scale bar 100 µm.

After reaching certain density, cells in the co-culture formed a dynamic network that could tear and regrow over the course of hours (Video S2). These data suggest that the cells that contribute to it are contractile. In the mouse testis the only two cell types that have this capacity are peritubular myoid cells and endothelial cells both of which express αSMA (Figure S1). Usually towards the end of the second week of culture different cell populations started to form networks of cells (Figure 6). There were strain- and age-dependent variations, however, generally in the co-cultures started from C57BL/6 and juvenile mice seminiferous tubules this phenomenon was observed somewhat earlier than in NMRI or FVB/N and adult-derived co-cultures. Alpha smooth muscle actin positive cells were observed on the edge of a retreating frontier of cells (Figure 6A), whereas slender matrices of WT1-expressing Sertoli cells were often located at areas of lower cell density (Figure 6B). These areas were also recruited into clusters or cord-like structures later on. Spermatogonial cells (MAGE-B4) were found single-dwelling or in clusters that were associated with the tightening networks of cells (Figure 6A and 6C).

**Figure 7:**
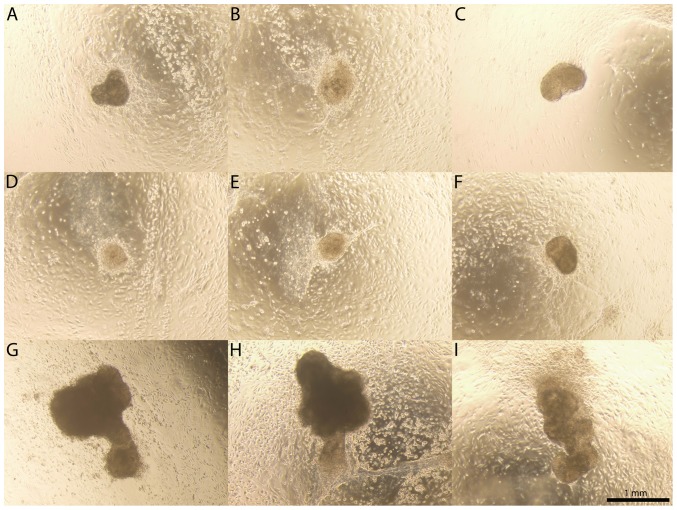
Collage of clusters. Cluster formation started to take place in the co-cultures right after gain of confluency and thereafter throughout 8 weeks. **A–F**) Co-cultures invariably gave rise to clusters of approximately 200–700 µm in diameter. **G–I**) Occasionally we also observed bigger clusters that were partly detached from the underlying co-culture and held back by a stalk of cells. Scale bar 1 mm.

Depending on the origin of the seminiferous tubule fragments (strain and age of the used animals) the spontaneous formation of clusters and cord-like structures appeared between 1.5 to 4 weeks of culture. There were no exceptions to this although the number, shapes and sizes of the clusters and cord-like structures varied from plate to plate. The clusters (roughly 200–700 µm in diameter; Figure 7A–F) were formed by two alternative mechanisms: they were either remnants of the seminiferous tubules that later on gathered cells around them (Video S3), or they coalesced from a seemingly homogenous matrix of cells (Video S4). Cluster formation could be accelerated by exposing the co-cultures to angiotensin II, an inducer of peritubular myoid cell contraction [40] (Videos S5, S6). Based on qRT-PCR, melting curve analysis and gel electrophoresis these clusters were formed by Sertoli cells (4/10 were positive for *WT1*, 2/10 for *Gdnf*), fibroblasts [4/10 positive for *Fsp1* (Fibroblast-specific protein-1)] [41], and peritubular myoid cells (9/10 positive for *αSMA*). This is in line with earlier findings showing that rodent Sertoli and peritubular cells form clusters of cells in a co-culture [42]. After 3–6 weeks of culture we occasionally observed spherical or slightly elongated clusters of cells also (in the range of 1–2 mm; Figure 7G–I, Figure S5). These were highly similar to the previously described protrusions formed during prolonged co-culture of juvenile rat Sertoli and peritubular myoid cells [43].

**Figure 8:**
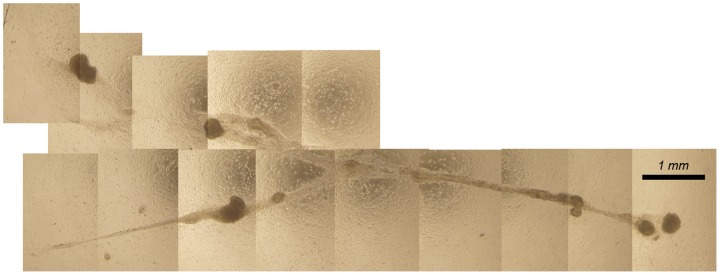
Phase-contrast image of a cord-like structure. First signs of cord-like structure formation were usually apparent after 3–4 weeks of culture. Cord-like structures sometimes branched and could reach a length of more than 10 mm.

Co-cultures invariably gave rise to cord-like structures that could be up to 15 mm of length (Figure 8). Formation of cord-like structures took place through compaction of cellular networks suggesting an active role for peritubular myoid cells in the process, and clusters seemed to coordinate this process (Videos S7, S8). The role of the clusters in the orchestration of cord-like structure formation was also supported by the fact that no formation of cord-like structures was observed when we used single cell suspensions of testis homogenate to establish co-cultures. Quantitative RT-PCR and gel electrophoresis showed that transcripts of Sertoli cell (*WT1*), peritubular myoid cell (*αSMA*), fibroblast (*Fsp1*) and spermatogonial cell (*c-Kit*, *CD9*, *Cip2a* and *Gfr1α*) markers were present in these structures (data not shown).

**Figure 9:**
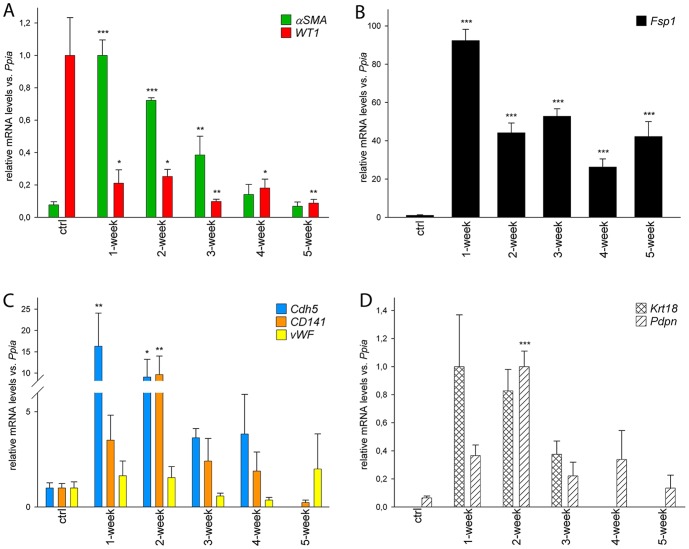
Quantitative RT-PCR analysis of somatic cell-specific marker expression in starting material and 1–5-week co-cultures. **A**) Relative *αSMA* mRNA levels were increased in the beginning of the culture, whereas *WT1* levels displayed the opposite. **B**) Fibroblast marker *Fsp1* was found at a very high level from week 1 onwards. **C**) Endothelial cell marker mRNA levels were acutely increased (*Cdh5* and *CD141*) or did not change (*vWF*) during the course of 5 weeks. **D**) Messenger-RNAs associated with juvenile, immature Sertoli cells (*Krt18* and *Pdpn*) were found at their highest level in 1–2 week co-cultures. n = 3, SEM; *, p<0.05; **, p<0.01, ***, p<0.001.

When the cord-like structure formation had completed the co-cultures remained in a relatively stagnant state (see Video S1, for instance). As shown above, αSMA-expressing cells played a central role in the formation of clusters and cord-like structures and the relatively homogenous culture outside the cord-like structures and clusters had a relatively small fraction of αSMA, WT1 or spermatogonia-associated marker-expressing cells (data not shown). To answer which cells did not get recruited into secondary structures but throve in the culture conditions, we analysed how different testicular somatic cell populations acted during the course of culture by qRT-PCR (Figure 9). We used *αSMA* to represent peritubular myoid cells, *WT1* for Sertoli cells, *Fsp1* as a testicular fibroblast marker, and *Cdh5* (Cadherin 5) [44], *CD141* [45] and *vWF* (von Willebrand factor) [46] to represent endothelial cells. The levels of *αSMA*, *Fsp1*, *Cdh5* and *CD141* mRNAs were elevated shortly after the beginning of the cultures but all except for *Fsp1* returned to control levels at later time points (Figure 9A–C). There were no statistically significant changes in the relative abundance of *vWF* mRNA (Figure 9C). *WT1* levels plummeted right after the beginning of the culture suggesting that the number of Sertoli cells did not change much while other somatic cell populations actively proliferated in the co-culture (Figure 9A). This is understandable since Sertoli cells in the adult are thought to be terminally-differentiated and quiescent cells. Because Sertoli cells in the co-culture probably have to adopt features that developmentally only immature, juvenile-type Sertoli cells possess, we analysed whether immature Sertoli cell-associated markers are enriched over the course of culture. Indeed, markers of immature Sertoli cells such as Krt18 ((Cyto)keratin-18) [47] and *Pdpn* (podoplanin) [48], were elevated shortly after the beginning of the culture at mRNA (Figure 9D) and protein level (Figure S6). As expected, *Krt18* mRNA levels in adult seminiferous tubules were undetectably low. These data suggest that adult-type mature Sertoli cells in the culture have to enter a dedifferentiation process to have an active role in secondary structure formation.

## Discussion

Due to the complexity of testicular architecture and function, novel *in vitro* approaches need to be developed to study the interaction of spermatogonia with testicular somatic cells. Even though SSCs can be maintained for extended periods of time *ex vivo*, co-culture with other stem cell niche contributors would provide the most physiologically relevant environment to study their biology. In the present study, a method for long-term co-culturing of spermatogonia with Sertoli and peritubular myoid cells was developed. Germ cells and somatic cells from small fragments of mouse seminiferous tubules proliferate *in vitro* and form a co-culture that can be maintained for at least five weeks. Since spermatogonia are able to survive and propagate for weeks and their life span is likely limited by Sertoli cells and not by the spermatogonia-intrinsic factors, we assume that stem cell niche-like conditions have been reconstructed *in vitro*. The advantages of this method are that no special isolation techniques and devices are required, no cell sorting is necessary, no exogenous growth factor stimulation is needed and it can be established in simple culture medium.

During the five-week culture expression of a wide range of spermatogonial markers was recorded. In adult-derived co-cultures great majority of differentiating cells was lost during the first week of culture. Taken together our results suggest that the conditions favour stem/progenitor spermatogonia but do not support differentiation of germ cells. Interestingly enough, a transcript for *Nanog*, a novel A-single spermatogonial marker [11], and rare Nanog-expressing cells were identified in the co-cultures. We also proved that the spermatogonia in our co-cultures remain able to enter spermatogenic differentiation if they are transplanted into *in vivo* settings. The presence of GFP-positive pachytene spermatocytes in mice depleted of germ cells clearly points at fully functional SSCs after a month of *ex vivo* co-culturing.

Sertoli cells secrete GDNF that is an important regulator of undifferentiated spermatogonia [37], [49]–[50], a factor that can be detected in our co-culture system at a comparable level as that used in SSC cultures in Sertoli cell-free conditions [6]. Additionally, it is highly likely that locally, i.e., in the interface between Sertoli cells and SSCs, the concentrations are even higher. *Gdnf* levels could be elevated by FSH, which shows that Sertoli cells were fully functional and responsive to physiological stimuli [51]. After the onset of spermatogenesis the dominant function of FSH on Sertoli cells shifts from driving their proliferation to nurturing germ cells via paracrine growth factor production [52]. Accordingly, FSH had a greater impact on one-week-old co-cultures established from adult than juvenile mice. Change in the paracrine milieu induced by FSH further activated SSCs as GDNF receptor *Gfr1α* mRNA expression was stimulated at the same time points. Our finding that FSH has a greater impact on the *Stra8* level in cultured juvenile than in adult testicular cells probably stems from the developmental state of these cells. Premeiotic/meiotic transition of the most advanced germ cells takes place on day 8–10 in mice [53] and therefore a relatively greater number of spermatogonia are bound to enter meiosis in juvenile than in adult mouse testis.

Sertoli cells have a crucial role in the development of testis cords in the fetal gonad [54]. In the present study we noticed that the formation of cord-like structures was centered around cell clusters that often expressed Sertoli cell markers. It has been shown by a number of research groups that testicular single cell homogenates from a juvenile rat spontaneously give rise to cord-like structures *in vitro* [42]–[43], [55]–[58]. Recently, using early postnatal mouse testis cells, Yokonishi and colleagues were able to reconstruct well-organized tubular structures *in vitro* [18]. We show here that adult mouse seminiferous tubule cells have the same capacity and they spontaneously give rise to cord-like structures *in vitro*. Interestingly, formation of these structures was observed to be associated with stimulation of immature Sertoli cell markers (Keratin-18 and *Podoplanin*) suggesting that adult, mature Sertoli cells may have to dedifferentiate to be able to support the development of secondary structures *in vitro*. Schlatt and co-workers have demonstrated that only early steps of testis tissue regeneration can take place *in vitro*. A change in the environment that is gained by grafting spherical cell aggregates into an immunodeficient mouse is required to enable further morphogenesis [57]. However, fairly good level or organization can be achieved by optimizing the culture conditions [18].

We describe here a novel method to study the interaction of Sertoli cells, myoid cells and spermatogonia *in vitro*. Seminiferous tubule cells spontaneously gave rise to a co-culture that could be maintained for weeks. Based on relatively high levels of GDNF in the culture medium and a wide range of spermatogonia present in the culture during the first five weeks of culture, we propose that this model could be used to study the interaction of testis stem cell niche components *in vitro*. Formation of secondary structures takes place spontaneously and in a highly repetitive manner possibly enabling research on early steps of testis development and regeneration *in vitro*.

## Materials and Methods

### Ethics statement

Turku University Committee on the Ethics of Animal Experimentation approved all animal experiments (permission no. 2008-03959).

### Establishing a two-dimensional co-culture of mouse seminiferous tubule cells

To establish a co-culture for mouse seminiferous tubule cells, we exploited a phenomenon described by Eddy and Kahri for rat seminiferous tubules [59]: spontaneous proliferation and migration of testicular cells out from small segments of seminiferous tubules *in vitro*. Male C57BL/6 and transgenic mice ubiquitously expressing eGFP [60]; purchased from The Jackson Laboratory (Bar Harbor, Maine, USA) and maintained in NMRI strain in Turku Center for Disease Modeling (TCDM, Turku, Finland)) were housed under environmentally controlled conditions (12 h light/12 h darkness; temperature, 21±1°C) at the Animal Centre of Turku University (Turku, Finland).

The mice were sacrificed by cervical dislocation under CO_2_ anaesthesia and the testes were collected. Under sterile conditions the testes were decapsulated and seminiferous tubules were dissected free from the interstitial tissue on a Petri dish in 15% inactivated fetal calf serum (iFCS; v/v) in DMEM/F12 (referred to hereafter as culture medium). Next the seminiferous tubules were cut into approximately 1-mm-long segments and 50–100 segments were moved onto cell culture plates with small amount of medium (100, 200 and 350 µl of suspension was plated onto 24-, 12-, and 6-wells plates, respectively). During 6–10 hours incubation [37°C, 5% (v/v) CO_2_, humidified atmosphere] the short fragments of seminiferous tubules attached on the bottom of the wells, and thereafter the amount of medium was increased. Culture medium was exchanged every two to three days. Originally we used seminiferous tubule segments from immature mice but noticed later that mice of different ages (from <10-day-old to 17-month-old) can be successfully used to establish the culture. The co-cultures were maintained for up to eight weeks as such and monitored by phase contrast microscopy, or samples were collected for experiments and analyses as described in the following sections.

### Immunocytochemistry

For immunocytochemistry, the co-cultures were established on uncoated or laminin-coated (20 µg/ml; L2020, Sigma Chemical Co., St. Louis, Mo, USA) (for less than 20-day cultures) coverslips. After the culture the cells on a coverslip were fixed with cold 4% (v/v) PFA (paraformaldehyde) for 10 minutes and stored in cold PBS (phosphate-buffered saline, pH 7.4) or TBS (Tris-buffered saline, pH 7.55). Fixed cells were washed three times 5 min in PBS/TBS, and then permeabilized with 0.1% (v/v) Triton X-100 in PBS/TBS for 10 min followed by three washes with PBS/TBS for 5 min each. Before primary antibody treatment the specimens were incubated (1 h, RT) in 10% (v/v) normal serum (NS) or bovine serum albumin (BSA) in PBS/TBS. The specimens were then incubated with rabbit polyclonal anti-WT1 (1∶500; C-19, sc-846, Santa Cruz Biotechnology Inc., Santa Cruz, CA, USA), polyclonal goat anti-Vimentin (1∶200; C-20, sc-7557, Santa Cruz), rabbit polyclonal anti-MAGE-B4 (1∶200; [24]), mouse monoclonal anti-α-smooth muscle actin (1∶500; A2547, Sigma), rabbit polyclonal anti-Nanog (1∶200; M-149, sc-33760, Santa Cruz), rabbit polyclonal antiphosphorylated-histone H3 (1∶500; #9701, Cell Signaling Technology, Danvers, MA, USA), goat polyclonal anti-PLZF (1∶500; AF2944, R&D Systems Inc., Minneapolis, MI, USA), goat polyclonal anti-LIN28 (1∶500; AF3757, R&D Systems), rabbit polyclonal anti-Ddx4 (1∶500; ab13840 Abcam, Cambridge, UK), mouse monoclonal anti-Ki-67 (1∶5000; M7240, DAKO, Glostrup, Denmark) or mouse monoclonal anti-Keratin-18 (1∶100; C-04, sc-51582, Santa Cruz) antibody overnight. The antibodies were diluted in 1% (v/v) NS/BSA in PBS/TBS. Negative controls lacked primary antibody.

After three washes with PBS/TBS 5 min each, the specimens were incubated with secondary antibody solution. The following secondary fluorescent antibodies were used: A11029, A11032, A11037, A11055, A11058, A21200, A31573 (diluted 1∶200 to 1∶2000 in 1% (v/v) NS/BSA in PBS/TBS; all purchased from Invitrogen, Carlsbad, CA, USA). After 1 hour of secondary antibody incubation (RT) the slides were washed three times with PBS/TBS and mounted with UltraCruz mounting medium (sc-24941, Santa Cruz Biotechnology Inc.). Images were captured using Olympus DP72 camera (Olympus Optical Co., Ltd, Tokyo, Japan) installed on Leica DMRBE microscope (Leica, Wetzlar, Germany), or Zeiss AxioImager M1 (Carl Zeiss MicroImaging GmbH, Heidelberg, Germany) coupled with a Zeiss AxioCam MR3 monochrome cooled-CCD camera and analyzed with Zen 2012 software. At least three parallel experiments were performed. For testis tissue sections, a protocol described previously was used [15] with slight modifications: for nuclear antigens the antigen retrieval step was performed in a pressure cooker (PickCell Laboratories, Amsterdam, the Netherlands).

### FSH and GDNF treatments on one-week-old co-cultures

To study the effect of different factors on 1-week co-culture, they were treated with recombinant human FSH (Gonal F, Serono, Geneva, Switzerland) or recombinant mouse glial cell-line derived neurotrophic factor (PeproTech, London, UK). FSH and GDNF were added to 15% (v/v) iFCS in DMEM medium (final concentration 10 ng/ml for both) that was supplemented with MIX (1-methyl 3-isobutyl xanthine, 0.2 mmol/l; Aldrich Chemie, Steinheim, Germany) in the FSH experiment. Samples were collected 6, 12, 24, 48 and 72 hours after treatment by scraping off the cells, pelleting them by centrifugation (1200 rpm, RT, 5 min) and snap-freezing them in liquid nitrogen. Additionally, in the FSH experiment the medium was exchanged after 72 hours and samples were collected 24 hours later. Within three independent experiments each treatment was applied on three parallel samples.

### Angiotensin II and LH/hCG analogue exposure to co-cultures

Angiotensin II has been shown to induce contraction of peritubular myoid cells *in vitro* [40]. We treated co-cultures at various time points with 100 nmol/l angiotensin II (A9525, Sigma) or vehicle (PBS) only and imaged and observed the co-culture by time-lapse imaging (one frame per 10 sec; see below for details).

LH/hCG analogue Pregnyl (Organon, Oss, the Netherlands) was administered in the culture medium of 2–3-week co-cultures at a final concentration of 10 ng/ml. Control samples got the vehicle only. Samples were collected 24 and 48 hours later (n = 4) and snap-frozen in liquid N_2_.

### RNA isolation and cDNA synthesis

RNA was isolated from the original suspension that was to be plated (control sample), cell pellets collected 1–5 weeks after the initiation of the culture, or cell pellets collected at certain time points after different treatments specified in other paragraphs of Materials and Methods. Trisure reagent (Bioline, London, UK) was used for total RNA isolation according to the manufacturer's instructions. After isolation, RNA concentration was measured using a NanoDrop device (ND-1000; NanoDrop Technologies, Wilmington, DE, USA) and the RNA sample was run on agarose gel to confirm good quality of the isolated RNA (intact 28S and 18S ribosomal RNA bands). One microgram of RNA was processed further. Firstly, traces of contaminating genomic DNA were removed by treating the samples with DNase I (Invitrogen, Carlsbad, CA, USA). DyNAmo SYBR Green 2-step qRT-PCR Kit (Finnzymes, Espoo, Finland) was used for cDNA synthesis and 0.5 µg of template RNA was reverse-transcribed in a 20-µl-reaction with oligo(dT) primers while another 0.5 µg was used as a template in RT- reaction.

### Whole transcriptome amplification

For samples of low RNA yield (clusters and cord-like structures) whole transcriptome amplification was performed using Qiagen Quantitect kit (207043; Qiagen, Hilden, Germany) according to the manufacturer's instructions. Complementary DNA obtained from the high yield reaction was diluted 1∶200 and used in qRT-PCR.

### Real-time PCR

With the help of Primer 3 software (http://frodo.wi.mit.edu/) and mRNA sequence data available at NCBI and Ensembl databases, primers were designed to be located to different exonic sequences (Table 2). For *Nanog* the primers lie within one exon. However, RT-minus reactions that were run in parallel never gave a positive signal. Amplification of target cDNAs was done using CFX96 real-time PCR detection system device (Bio-Rad Laboratories Inc., Hercules, CA, USA) and the DyNAmo Flash SYBR green qPCR kit (F-415L; Finnzymes, Espoo, Finland) according to the manufacturers' instructions. Quantitative real-time PCR was performed under the following conditions: 95°C for 7 min followed by 40 cycles of 94°C for 1 s and 55–64°C (depending on the primer pair; see Table 2) for 15 s. Relative gene expression data was normalized to transcript levels of *Ppia* (cyclophilin A) and *L19* (L19 ribosomal protein) using 2∧−ΔΔC(t) method [61]. PCR specificity was verified by gel electrophoresis and melting curve analysis, one band of the expected size and a single peak, respectively, were required.

**Table 2:** Primer design, annealing temperatures and PCR product lengths of the studied mRNAs.

Gene	Accession number	Annealing temperature	Primers	Product length
*αSMA*	NM_007392	62°C	5′-TGCTGTCCCTCTATGCCTCT-3′ 5′-GAAGGAATAGCCACGCTCAG-3′	184 bp
*CD141*	NM_009378	55°C	5′- TCTGCGAGCATTTTTGTGTC-3′ 5′-GCACTCTCCATCCACCAACT-3′	215 bp
*CD9*	NM_007657	56°C	5′-TGCAGTGCTTGCTATTGGAC-3′ 5′-GGCGAATATCACCAAGAGGA-3′	219 bp
*Cdh5*	NM_009868	62°C	5′-ATTGAGACAGACCCCAAACG-3′ 5′-TTCTGGTTTTCTGGCAGCTT-3′	238 bp
*CIP2A*	NM_172616	57°C	5′-GCGCCATGTACTCAGTCAGA-3′ 5′-AGGAAGCAGAAGGGTCACAA-3′	234 bp
*c-Kit*	NM_021099	56°C	5′-ATCCCGACTTTGTCAGATGG-3′ 5′-AAGGCCAACCAGGAAAAGTT-3′	192 bp
*Erm*	NM_023794	57°C	5′-CAAGAGCCCCGAGATTACTG-3′ 5′-CTCGGGTACCACGCAAGTAT-3′	146 bp
*Fsp1*	NM_011311	64°C	5′-ACTCAGGCAAAGAGGGTGAC-3′ 5′-TGCAGGACAGGAAGACACAG-3′	188 bp
*Gdnf*	NM_010275	61°C	5′-CGGACGGGACTCTAAGATGA-3′ 5′-CGTCATCAAACTGGTCAGGA-3′	209 bp
*Gfr1α*	NM_010279	63°C	5′-TGTACTTCGTGCTGCCACTC-3′ 5′-GCTGAAGTTGGTTTCCTTGC-3′	166 bp
*Gpr125*	XM_991709	59°C	5′-GACCTGACGAACAACCGAAT-3′ 5′-CTGGTGTCTCGCACAGTGAT-3′	235 bp
*Krt18*	NM_010664	55°C	5′-CGAGGCACTCAAGGAAGAAC-3′ 5′-CTTGGTGGTGACAACTGTGG-3′	246 bp
*L19*	NM_009078	55°C	5′-GGACAGAGTCTTGATGATCTC-3′ 5′-CTGAAGGTCAAAGGGAATGTG-3′	195 bp
*Nanog*	NM_028016	64°C	5′-CCAGTGGAGTATCCCAGCAT-3′ 5′-GAAGTTATGGAGCGGAGCAG-3′	236 bp
*Pdpn*	NM_010329	58°C	5′-GCCAGTGTTGTTCTGGGTTT-3′ 5′-AGAGGTGCCTTGCCAGTAGA-3′	191 bp
*PLZF*	NM_001033324	59°C	5′-AACGGTTCCTGGACAGTTG-3′ 5′-CCCACACAGCAGACAGAAGA-3′	172 bp
*Ppia*	NM_008907	63°C	5′-CATCCTAAAGCATACAGGTCCTG-3′ 5′-TCCATGGCTTCCACAATGTT-3′	164 bp
*Stra8*	NM_009292	57°C	5′-ATGCAATGTTGCTGAAGTGC-3′ 5′-GGAAGCAGCCTTTCTCAATG-3′	161 bp
*Sycp3*	NM_011517	60°C	5′-AGCCAGTAACCAGAAAATTGAGC-3′ 5′-CCACTGCTGCAACACATTCATA-3′	106 bp
*vWF*	NM_011708	63°C	5′-GGCAAGAGAATGAGCCTGTC-3′ 5′-AAGCCAAAGGTCTCACTGGA-3′	172 bp
*WT1*	NM_144783	64°C	5′-AGGTTTTCTCGCTCAGACCA-3′ 5′-GCTGAAGGGCTTTTCACTTG-3′	160 bp

### GDNF enzyme-linked immunosorbent assay (ELISA) and testosterone radioimmunoassay

Culture medium samples were collected on a weekly basis and stored at −80°C. GDNF concentration in the medium was determined using a mouse glial cell-line derived neurotrophic factor ELISA kit (CSB-EL009356MO, Cusabio Biotech Co., Ltd, Wuhan, China) according to the manufacturer's instructions. Testosterone levels in the culture media were measured using Spectria testosterone RIA (radio-immunoassay; Orion Diagnostica, Espoo, Finland).

### Flow cytometry (FACS)

Cells were scraped off the bottom of the plates into a small volume of medium, and moved to 4 ml of prewarmed culture medium containing collagenase/dispase (0,5 mg/ml; Roche, Indianapolis, IN, USA), collagen IV (0,5 mg/ml; Worthington Biochemical, Freehold, NJ, USA), hyaluronidase (1 mg/ml; Sigma) and DNase I (0,04 mg/ml; Sigma). The samples were incubated at 37°C for 25 min in horizontal rotation. Thereafter cells were pelleted by centrifugation (1500 rpm, 5 min) and the supernatant was removed. The cells were resuspended in lysis buffer (PBS+2% Triton X-100) containing propidium iodide (5 µg/ml; P4864, Sigma) and incubated 15 min at +4°C in horizontal rotation. Thereafter the nuclei were pelleted and resuspended to 500 µl of PBS. The solution was passed through 100 µm cell strainer and analysed by FACS. Determination and analysis of the cell populations in the suspension were done using FACSCalibur flow cytometer, Cell Quest software (BD Biosciences, Mansfield, MA, USA) and Flowing-Software Version 2.5.0 (Turku Center for Biotechnology, Turku, Finland).

### Transplantation of cells to rete testis

Adult C57BL/6 mice were rendered sterile by treating them with busulfan (40 mg/kg, i.p.). One-month after the injection approximately 100 000 cells isolated from 1-month-old adult ubiquitously GFP-expressing mouse seminiferous tubule fragment-derived cultures were transplanted via the rete testis according to a protocol described previously [36]. Single cell suspension for this purpose was prepared as described above for FACS. Five weeks post-operatively the testes were dissected and seminiferous tubules were imaged with a Zeiss SteREO Lumar V12 microscope with eGFP bandpass filter (Carl Zeiss MicroImaging, Thornwood, NY, USA).

### Time-lapse imaging

During live-cell imaging the samples were maintained in an incubator at 37°C and 5% CO2, humidified atmosphere. Frames were captured at defined intervals (5 sec to 30 min) using Olympus DBH1 camera attached to a Olympus IX71 microscope with 10X phase-contrast objective. All images were acquired digitally using Cell R software (Olympus).

### Statistical analysis

The results were analyzed for statistically significant differences using one-way analysis of variance, followed by Tukey's test for multiple comparisons of independent groups of samples. An independent samples t-test was used for statistical analyses of pair-wise comparisons. The *p* values less than 0.05 were considered statistically significant.

## Supporting Information

Figure S1 **Validation of the used antibodies.** Formalin-fixed, paraffin-embedded adult mouse testis tissue sections were stained with the same antibodies that were also used for co-cultures. Positive control staining for **A**) αSMA (red), **B**) MAGE-B4 (red), **C**) WT1 (red) and **D**) Vimentin (red). DAPI stains the nuclei blue. Insets represent negative control stainings.
(TIF)

Figure S2 **Nanog staining for ESCs.** To validate the functionality of the used Nanog antibody, we stained mouse embryonic stem cells side-by-side with sc-33760 (top panel) and ab80892 (middle panel) (Abcam Inc., rabbit polyclonal anti-mouse Nanog antibody). The antibodies gave identical result. Negative control staining is in the bottom panel. M, murine feeder cell (mouse embryonic fibroblast). Scale bars: top and middle panel 10 µm, bottom panel 20 µm.
(TIF)

Figure S3 **Proliferation of germ cells in co-cultures.** Double-immunofluorescent staining for 1-week-old co-culture showed that some Ddx4 (green) positive germ cells also expressed proliferating cell antigen Ki-67 (red). DAPI stains nuclei blue. Scale bar 20 µm.
(TIF)

Figure S4 **FSH treatment indirectly affected mRNA levels of spermatogonial markers.** Steady state levels of *Gfr1α* mRNAs were acutely increased by FSH treatment in co-cultures established from **A**) adult and **B**) juvenile mouse seminiferous tubules. **C**) FSH did not consistently affect *Stra8* levels in adult-derived co-cultures, **D**) whereas they were uniformly upregulated by the treatment in juvenile-derived co-cultures. FSH elevated **E**) *c-Kit*, **F**) *Gpr125* and **G**) *PLZF* mRNA levels in 1-week adult-derived co-cultures. White bars, control; black bars, 10 ng/ml rhFSH; n = 3, SEM; *, p<0.05; **, p<0.01; *** p<0.001.
(TIF)

Figure S5 **Haematoxylin-eosin-stained cross-section of a cluster that exhibits relatively high degree of bilateral symmetry.** Clusters that had a diameter of 1–2 mm were only partially connected to the underlying co-culture and moved back and forth when the medium was exchanged. These structures were slightly disorganized but occasionally displayed relatively high level of symmetry.
(TIF)

Figure S6 **Immunocytochemical staining for 4-week co-culture showing the presence of Vimentin (green) and Keratin-18 (red) positive cells side-by-side.** Vimentin and Keratin-18 only colocalize (orange) at areas where the cells are in a physical contact. DAPI stains the nuclei of cells (blue).
(TIF)

Video S1 **Eight-week follow-up of co-culture. One frame per every 1–4 days.**
(MOV)

Video S2 **Dynamic nature of confluent co-cultures.** One frame per 20 minutes; total 45 hours.
(MOV)

Video S3 **Formation of a cluster by attraction of cells at a near distance to a seminiferous tubule remnant.**
(MOV)

Video S4 **Formation of a cluster by coalescence of seemingly homogenous matrix of cells taking place in the lower right corner.**
(MOV)

Video S5 **Angiotensin II-induced contraction of cells in the co-culture followed 5 minutes after the exposure.** One frame per 5 seconds; total 5 min.
(MOV)

Video S6 **Vehicle-treated (PBS) control co-culture that is followed three minutes after addition of vehicle.** One frame per 5 seconds; total 3 min.
(MOV)

Video S7 **Long-term follow-up of cord-like structure formation.** One frame per every 1–4 days.
(MOV)

Video S8 **Short-term follow-up of cord-like structure formation.** One frame per 12 minutes; total 29 hours.
(MOV)
