# Supplementary figures and images for: Reconstruction of Mouse Testicular Cellular Microenvironments in Long-Term Seminiferous Tubule Culture
Source: PLoS One. 2014 Mar 11;9(3):e90088. doi: 10.1371/journal.pone.0090088 (PMC3949678; doi:10.1371/journal.pone.0090088)

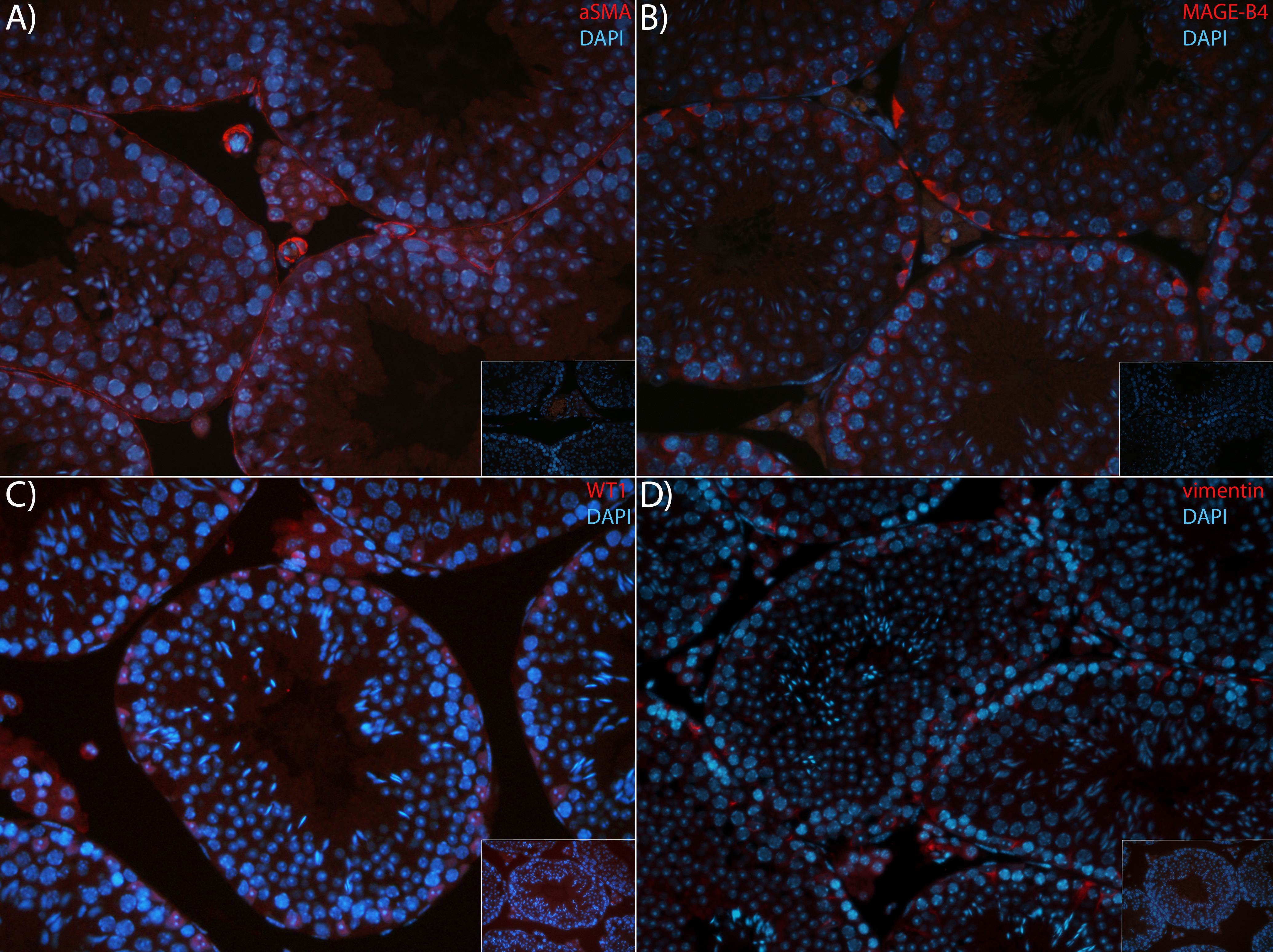

Supplement: Figure S1 — Validation of the used antibodies. Formalin-fixed, paraffin-embedded adult mouse testis tissue sections were stained with the same antibodies that were also used for co-cultures. Positive control staining for A) αSMA (red), B) MAGE-B4 (red), C) WT1 (red) and D) Vimentin (red). DAPI stains the nuclei blue. Insets represent negative control stainings. (TIF) [file pone.0090088.s001.tif]

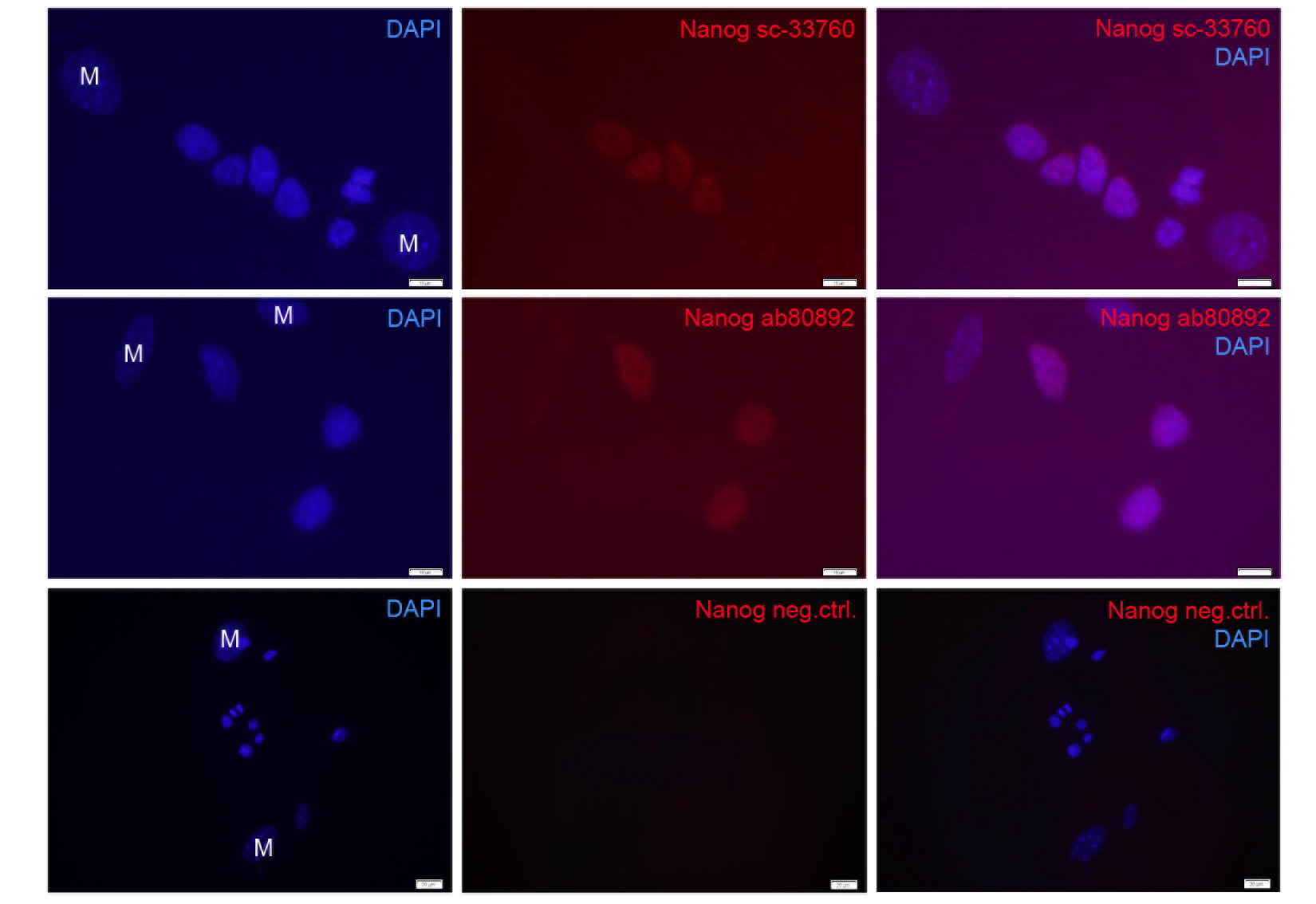

Supplement: Figure S2 — Nanog staining for ESCs. To validate the functionality of the used Nanog antibody, we stained mouse embryonic stem cells side-by-side with sc-33760 (top panel) and ab80892 (middle panel) (Abcam Inc., rabbit polyclonal anti-mouse Nanog antibody). The antibodies gave identical result. Negative control staining is in the bottom panel. M, murine feeder cell (mouse embryonic fibroblast). Scale bars: top and middle panel 10 µm, bottom panel 20 µm. (TIF) [file pone.0090088.s002.tif]

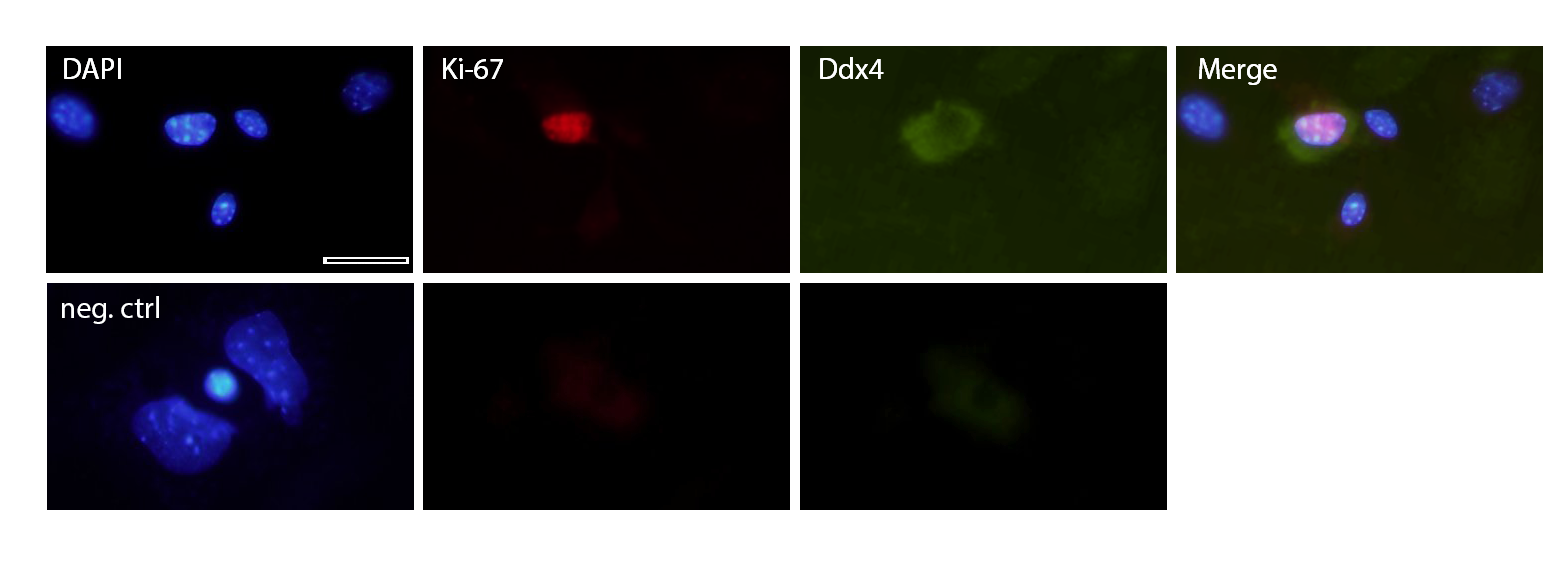

Supplement: Figure S3 — Proliferation of germ cells in co-cultures. Double-immunofluorescent staining for 1-week-old co-culture showed that some Ddx4 (green) positive germ cells also expressed proliferating cell antigen Ki-67 (red). DAPI stains nuclei blue. Scale bar 20 µm. (TIF) [file pone.0090088.s003.tif]

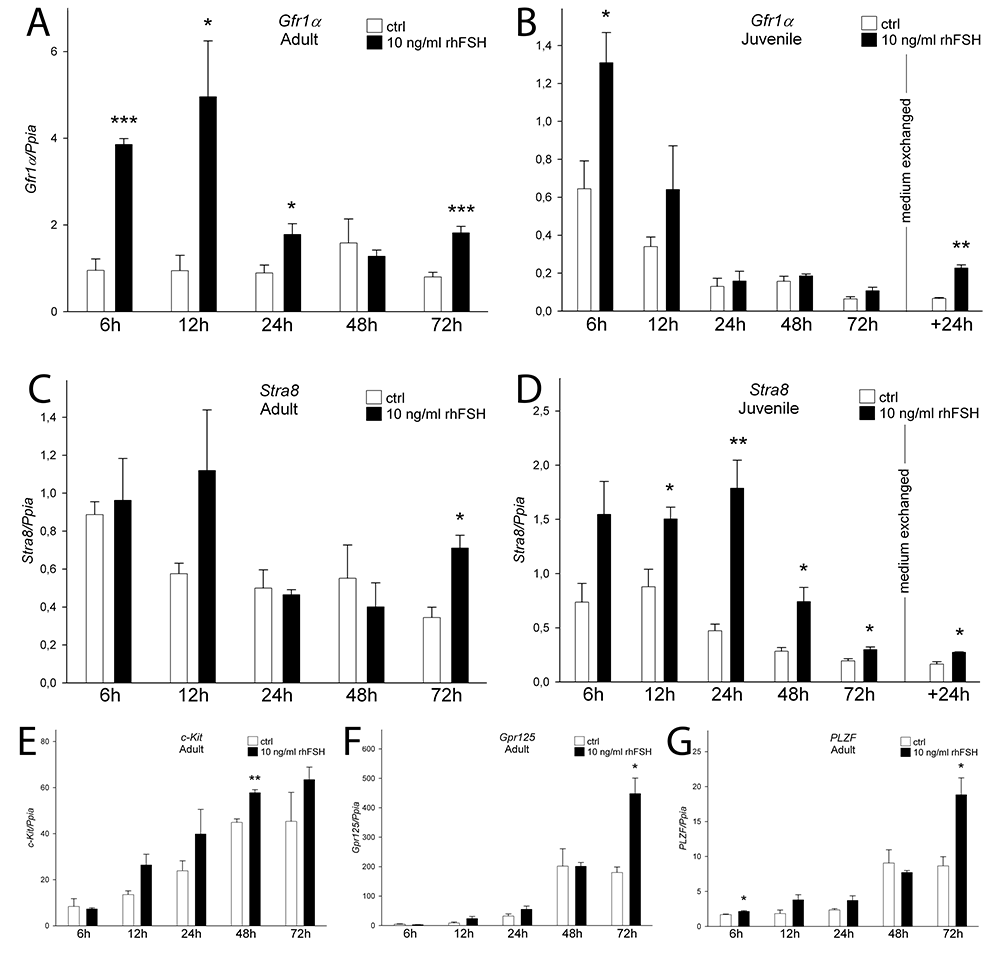

Supplement: Figure S4 — FSH treatment indirectly affected mRNA levels of spermatogonial markers. Steady state levels of Gfr1α mRNAs were acutely increased by FSH treatment in co-cultures established from A) adult and B) juvenile mouse seminiferous tubules. C) FSH did not consistently affect Stra8 levels in adult-derived co-cultures, D) whereas they were uniformly upregulated by the treatment in juvenile-derived co-cultures. FSH elevated E) c-Kit, F) Gpr125 and G) PLZF mRNA levels in 1-week adult-derived co-cultures. White bars, control; black bars, 10 ng/ml rhFSH; n = 3, SEM; *, p<0.05; **, p<0.01; *** p<0.001. (TIF) [file pone.0090088.s004.tif]

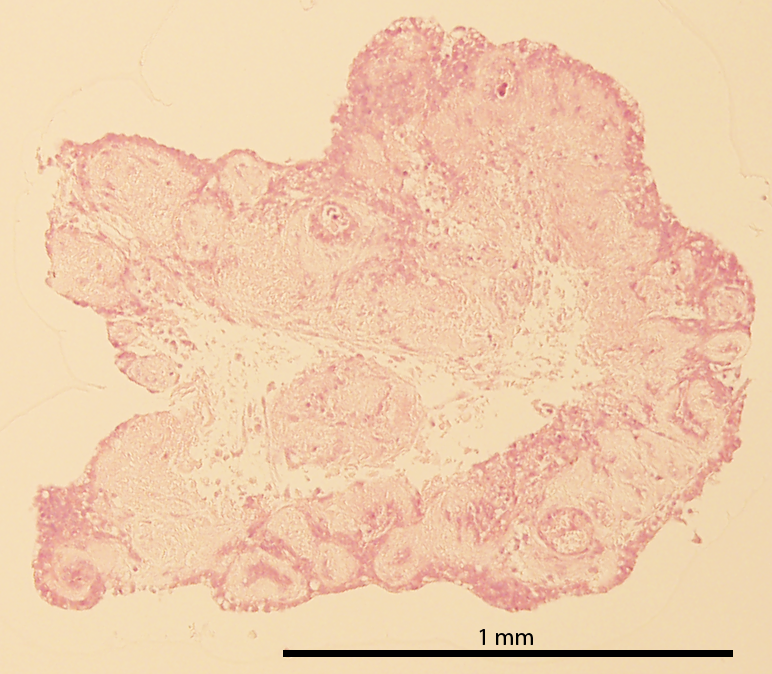

Supplement: Figure S5 — Haematoxylin-eosin-stained cross-section of a cluster that exhibits relatively high degree of bilateral symmetry. Clusters that had a diameter of 1–2 mm were only partially connected to the underlying co-culture and moved back and forth when the medium was exchanged. These structures were slightly disorganized but occasionally displayed relatively high level of symmetry. (TIF) [file pone.0090088.s005.tif]

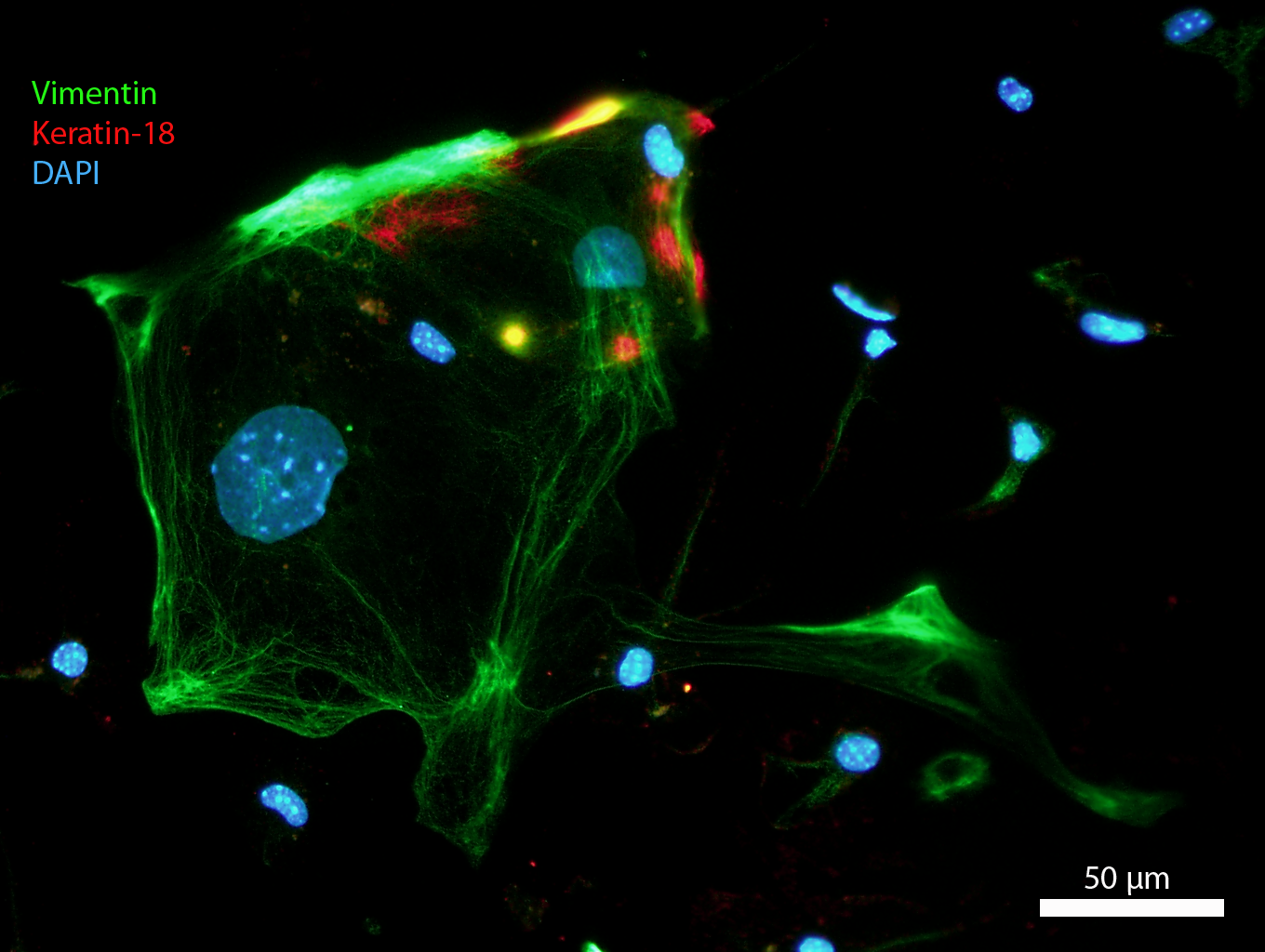

Supplement: Figure S6 — Immunocytochemical staining for 4-week co-culture showing the presence of Vimentin (green) and Keratin-18 (red) positive cells side-by-side. Vimentin and Keratin-18 only colocalize (orange) at areas where the cells are in a physical contact. DAPI stains the nuclei of cells (blue). (TIF) [file pone.0090088.s006.tif]
